# Prevalence and risk indicators of gingivitis and periodontitis in a Multi-Centre study in North Jordan: a cross sectional study

**DOI:** 10.1186/1472-6831-12-1

**Published:** 2012-01-03

**Authors:** Khansa Taha Ababneh, Zafer Mohammad Faisal Abu Hwaij, Yousef S Khader

**Affiliations:** 1 Periodontology, Department of Preventive Dentistry, Faculty of Dentistry, Jordan University of Science and Technology, Irbid, Jordan; 2 Community Medicine & Public Health, Faculty of Medicine, Jordan University of science and Technology, Irbid, Jordan

**Keywords:** Prevalence, Odds Ratio, Aggressive Periodontitis, Chronic Periodontitis, Gingivitis, Jordan

## Abstract

**Background:**

There are limited data about the epidemiology and risk factors/indicators of gingivitis, aggressive periodontitis (AgP) and chronic periodontitis (CP) in Jordan. The aim of this study was to assess the prevalence and risk indicators of gingivitis, AgP and CP.

**Methods:**

A sample of 595 subjects was randomly selected from subjects escorting out-patients attending a Medical Center, a Dental Teaching Hospital, and 2 private dental clinics. The socio-demographic variables, oral hygiene habits, income, smoking and Body Mass Index (BMI) were recorded. Full mouth periodontal examination was performed, and radiographs were taken for sites with probing depth > 3 mm.

**Results:**

About 76% had gingivitis, 2.2% had AgP and 5.5% had CP. Periodontitis was more frequent among males than females with a M: F ratio of 1.6:1 and the prevalence increased with age. Subjects who reported not using a tooth brush, smokers and subjects with BMI > 30 kg/m^2 ^had significantly higher prevalence of periodontitis. The risk for periodontitis was greater among subjects who reported positive family history and subjects with ≤ 12 years of education.

**Conclusions:**

This is the first study to report on the prevalence of gingivitis, CP and AgP in North Jordanian. Age, low education, low frequency of tooth brushing and family history were significantly associated with increased risk of periodontitis.

## Background

Periodontitis is a group of inflammatory diseases affecting the supporting tissues of the tooth. The American Academy of Periodontology (AAP) has classified periodontitis into aggressive periodontitis (AgP), chronic periodontitis (CP) and periodontitis as a manifestation of systemic diseases [1]. Both AgP and CP have a multi-factorial etiology with dental plaque as the initiating factor [2]. However, the initiation and progression of periodontitis are influenced by other factors including microbiologic, social and behavioral, systemic and genetic factors [3].

The prevalence of periodontal diseases varies in different regions of the world according to the definition of periodontitis and study population, and there are indications that they may be more prevalent in developing than in developed countries [4,5]. The National Health and Nutrition Examination Survey III (NHANES III) conducted in the United States (USA) between 1988-1994 [6] has demonstrated that 50% of the adult population has gingival inflammation. A national survey in the US has estimated that 19.9% of subjects aged 30 years, and 7.3% of those aged 90 years had clinical attachment level (CAL) ≥ 5 mm and 7 mm, respectively. A national survey in the United Kingdom estimated that 42% of 35 - 44 years old and 70% of 55-64 years old had CAL > 3.5 mm, indicating presence of periodontitis [7]. The prevalence of AgP in the US ranges between 0.6% in Whites and 2.6% in African-Americans [8]. The aims of this study are based on the fact that, to date, there is no information on the prevalence of these two diseases in Jordan and no controlled studies of the risk indicators of periodontitis in the country. Therefore, this is the first report on the prevalence of AP, CP and gingivitis and the first controlled study on their risk indicators in the northern part of Jordan.

## Methods

### Study Population

This investigation was undertaken with the understanding and consent of each participating subject and has been conducted in full accordance with ethical principles of the World Medical Association Declaration of Helsinki http://www.wma.net/en/30publications/10policies/b3/index.html. This study was approved by the Ethical Committee in the Deanship of Scientific Research at JUST and informed consents were obtained from the participants and the parents of those under 18 years of age, prior to the commencement of the study. The study included a random sample of people escorting out-patients attending JUST Medical Health Center and escorts to dental patients attending the Dental Department in Princess Basma Teaching Hospital and two dental private clinics in Irbid; no dental or medical patients were included. Princess Basma Teaching Hospital is a Ministry of Health hospital. The Ministry of Health is the main provider of primary health care (PHC) services in Jordan [9]. The reason for recruiting this population was based on the observation that subjects who accompanied patients in these centers are a diverse group in terms of residency, age and socioeconomic status (SES). Previous published work on the prevalence of periodontal disease and obesity among adults in north Jordan has used the same methodology [10].

Jordan has a population of around 6 million people. Irbid is the second largest populated governorate in Jordan with a population of 700,000. Although there are health centers and hospitals in the rural areas and in smaller cities in Irbid governorate, many people residing there frequently seek medical and dental treatment in Irbid city. Therefore, the sample of the study is believed to be representative of the population in the northern part of Jordan. The minimum sample size needed was calculated assuming that the prevalence of AgP is 4% among people aged 25 years or more. At a confidence level of 95%, power of 80%, and margin error of 2%, the minimum sample size was calculated as 481 subjects.

### Exclusion criteria

Subjects with ongoing or previous orthodontic treatment, subjects under current or previous periodontal treatment, those under the age of 14 years, pregnant women, subjects with diabetes mellitus or any syndromes, patients attending the medical/dental centers for dental or medical treatment, and subjects with any known/diagnosed form of immunosuppression or immunosuppressive medication were excluded from the study.

### Interview and Clinical Examination

A structured questionnaire was completed for each subject by one of 3 qualified dentists. It included information about sociodemographic factors, oral hygiene habits, frequency of dental visits, smoking and family history of periodontal diseases.

For each participant, full mouth periodontal examination was performed and recorded on a special examination form by one of the 3 examiners. These data included probing depth (PD), clinical attachment level (CAL), the gingival index (GI) of Löe and Silness,[11] and the plaque index (PI) of Silness and Löe [12]. Each tooth, except third molars, was examined by "walking" the periodontal probe around the whole circumference of the tooth. PD and CAL were measured at six sites per tooth (mesio-, mid-, and disto-buccal; mesio-, mid-, and disto-lingual/palatal). Inter-rate variability between the three examiners in the average, PD, CAL and GI were examined using one way Anova. There was no significant difference in the GI, PD and CAL between the three rates. Furthermore, the three rates similarly diagnosed periodontal diseases (AgP, CP). Intra-oral radiographs were taken for any site with PD > 3 mm (bitewing for posterior teeth and periapical radiographs for anterior teeth) to reach or confirm diagnosis. The body mass index (BMI) was calculated by the equation: Weight kg/length m^2^. The weight and length were self reported. The categorization of BMI was based on the WHO criteria that classify BMI < 25 kg/m^2 ^as "normal", BMI 25-29.9 kg/m^2 ^as "overweight" and BMI ≥ 30 kg/m^2 ^as "obese".

### Diagnosis

Periodontal health was defined as the complete absence of gingivitis and/or periodontitis at any site. Gingivitis was defined as the presence of signs of plaque-induced gingival inflammation in at least one site. Signs of gingival inflammation including redness, swelling and/or bleeding on probing, that were not attributable to any other cause. Subjects were classified as having gingivitis (only) if they did not demonstrate any sign of attachment loss, such as pockets, gingival recession and bone loss. In cases where the probing depth was greater than 3 mm, radiographs (bitewing for the posterior and periapical for the anterior teeth) were taken to investigate the presence of bone loss and, therefore, to differentiate between pseudopockets and true pockets. Periodontitis was defined as the presence of CAL > 2 mm on more than one tooth. The presence of alveolar bone loss was used to confirm the diagnosis of periodontitis. To differentiate between CP and AgP, clinical findings including PD, CAL, severity and (to a lower extent) pattern of bone loss, were used as diagnostic criteria. When the presence of CAL > 2 mm in addition to alveolar bone loss around at least two teeth was observed, one of which was a first molar, or when attachment and bone loss were present around first molars and/or incisors relatively earlier in life (i.e. < 50 years), especially where the characteristic arc-shaped defect(s) was/were observed, the case was diagnosed as AgP. Inconsistence between the amount of deposits and the amount of destruction, and the presence of family history further confirmed the diagnosis of AgP. On the other hand, CP was diagnosed when CAL > 2 mm and/or alveolar bone loss were observed in more than one tooth, usually (but not always) in older age groups, i.e. > 50 years. Young individuals with slight attachment and bone loss, where the amount of deposits was consistent with the amount of destruction, were diagnosed as having CP.

### Statistical Analysis

Statistical analysis was conducted using the Statistical Package for Social Sciences (SPSS version 15, Chicago, Illinois). Categorical variables were described using frequencies and percentages and continuous variables were described using means and standard deviations. Chi-square test was used to compare between percentages. The extent and severity of periodontal disease were analyzed using the General Linear Model multivariate procedure (GLM-multivariate analyses model). Binary logistic regression was used to determine factors associated with periodontal disease. A p-value of < 0.05 was considered statistically significant.

## Results

### Prevalence of Periodontal Diseases

Table 1 shows that overall, 75.8% of the study sample had gingivitis, 5.5% had CP, 2.2% had AgP and only 16% had clinically healthy periodontium. Most participants were 20-29 years old. Table 1 demonstrates that CP was not common among subjects aged < 40 years (2.3%). Above this age, the prevalence sharply increased to 21.2% in the group aged 40-49 years, and to 53.3% in subjects ≥ 50 years old. It was decided to sum AgP and CP together in one "periodontitis group" when studying risk indicators, because the number of AgP patients was too low for examining risk indicators.

**Table 1:** Prevalence of Gingivitis, CP and AgP According to Age

Age (years)	Diagnosis (Number)				
	***Healthy (%)***	***Gingivitis (%)***	***AgP (%)***	***CP (%)***	***Total (%)***

**< 20**	18 (20.0)	72 (80.0)	0 (0.0)	0 (0.0)	**90 (15.1)**

**20-29**	66 (21.3)	237 (76.5)	6 (1.9)	1 (0.3)	**311 (52.3)**

**30-39**	6 (6.1)	85 (86.7)	5 (5.51)	2 (2.0)	**98 (16.5)**

**40-49**	7 (10.6)	43 (65.2)	2 (3.0)	14 (21.2)	**66 (11.1)**

**≥ 50**	0 (0.0)	14 (46.7)	0 (0.0)	16 (53.3)	**30 (5.01)**

**Total**	**97 (16.3)**	**451 (75.8)**	**13 (2.2)**	**33 (5.5)**	**595**

### Participants' characteristics, smoking, oral hygiene habits and frequency of dental visits

This study included 595 participants (236 males and 359 females) aged 14 to 67 years with a mean (SD) of 28 (10.1) years. Their sociodemographic and relevant characteristics appear in Table 2, which shows that the majority of participants were females, had a family income ≥ 400 Jordanian Dinars (JOD), had > 12 years of education, lived in urban areas, had a normal weight, attended dental clinics on emergency only, reported regular tooth brushing, were non-smokers and reported no family history of periodontal diseases.

**Table 2:** Risk Indicators of Gingivitis and Periodontitis

Variables		Healthy	Gingivitis	Periodontitis	Total	p-value*	p-value^†^
		**N (%)**	**N (%)**	**N (%)**	**N (%)**		

**Gender**	*Male*	23 (9.7)	193 (81.8)	20 (8.5)	**236 (39.7)**		
	*Female*	74 (20.7)	258 (72.1)	26 (7.3)	**359 (60.3)**		
**Income **(JOD)^‡^	*< 400*	28 (9.5)	237 (80.6)	29 (9.9)	**295 (49.6)**	< 0.0005	< 0.0005
	*≥ 400*	69 (23.0)	214 (71.3)	17 (5.7)	**300 (50.4)**		
**Education **(Years)	*≤ 12*	21 (11.1)	135 (71.4)	33 (17.5)	**189 (31.8)**	0.101	< 0.0005
	*> 12*	76 (18.8)	316 (78.0)	13 (3.2)	**406 (68.2)**		
**Residency**	*Urban*	65 (18.5)	264 (75)	23 (6.5)	**352 (59.2)**	0.122	0.051
	*Rural*	32 (13.2)	187 (77.3)	23 (9.5)	**243 (40.8)**		
**BMI **(Kg/m^2^)^§^	*< 25*	78 (20.9)	280 (74.9)	16 (4.3)	**375 (62.9)**	0.002	< 0.0005
	*25-29*	16 (9.7)	130 (78.8)	19 (11.5)	**165 (27.8)**		
	*≥ 30*	3 (5.6)	40 (74.1)	11 (20.4)	**54 (9.3)**		
**Dental visits**	*Regular*	28 (29.5)	60 (63.2)	7 (7.4)	**95 (16.0)**	0.0005	0.070
	*On need*	40 (17.0)	178 (75.5)	17 (7.2)	**236 (39.7)**		
	*Emergency*	29 (11.0)	213 (80.7)	22 (8.3)	**264 (44.3)**		
**Brushing**	*No brushing*	1 (1.7)	53 (91.0)	4 (6.9)	**58 (9.7)**	< 0.0005	< 0.0005
	*Irregular(< 1/day)*	17 (9.8)	138 (79.3)	19 (10.9)	**174 (29.2)**		
	*Regular (≥ 2/day)*	79 (21.8)	260 (71.0)	23 (6.4)	**363 (61.1)**		
**Smoking**	*No*	88 (18.3)	357 (74.4)	35 (7.3)	**481 (81.1)**	0.018	0.027
	*Past*	1 (5.9)	13 (76.5)	3 (17.6)	**17 (2.9)**		
	*Current*	7 (7.4)	80 (84.2)	8 (8.4)	**95 (16.0)**		
**Family history**	*Yes*	22 (13.2)	126 (75.4)	19 (11.4)	**167 (28.2)**	0.304	0.024
	*No*	74 (17.5)	323 (76.2)	27 (6.4)	**425 (71.8)**		

*Gingivitis vs. Healthy

^† ^Periodontitis vs. Healthy

^‡ ^Jordanian Dinars

^§ ^Body Mass Index

### Risk Indicators of Periodontitis

The distribution of gingivitis and periodontitis according to sociodemographic and relevant characteristics is shown in Table 2. Periodontitis was more prevalent among males than females with a ratio of 1.6:1. A higher prevalence of periodontitis (9.9%) was noticed among subjects with a total family income < 400 JOD than among subjects with a higher income (≥ 400 JDs); the same applied to gingivitis. There was a significant difference in the prevalence of periodontitis according to income between periodontitis and healthy groups (*p = 0.0005*), as well as between the gingivitis and healthy groups. The majority of subjects with ≤ 12 years of education had gingivitis followed by periodontitis, whereas the majority of subjects with > 12 years of education had gingivitis and only 3.2% had periodontitis. A higher prevalence of gingivitis and periodontitis was found among subjects from rural areas than among those from urban areas. There were no significant differences in the prevalence of gingivitis or periodontitis according to residency, when compared to the healthy group (*p = 0.051*). As for BMI, it appeared that a significant difference exists between gingivitis and periodontally-healthy individuals as only 4% of subjects with BMI < 25 kg/m^2 ^had periodontitis, whereas 20.4% with BMI > 30 kg/m^2 ^had periodontitis (*p = 0.0005*). Subjects who reported emergency dental visits had a higher prevalence of gingivitis (80.7%) and periodontitis (8.3%) than subjects who reported regular dental visits.

The lowest prevalence of gingivitis (71%) and periodontitis (6.4%) was noticed among subjects who reported regular toothbrushing. In subjects reporting no brushing, the prevalence of gingivitis and periodontitis was almost 91% and 7%, respectively. The PI was stratified into 3 groups (PI < 1; PI = 1-2 and PI > 2) (results not shown in table). Within the AgP group 69.2% of individuals had PI 1-2 and 30.8% had PI > 2. Among CP subjects only 1 subject (3%) had PI < 1, 72.7% had PI 1-2 and 24.3% had PI > 2. Among the gingivitis group 15.3% had PI < 1, 61% had PI 1-2 and 23.7% had PI > 2. Within the healthy group 82.5% had PI < 1, 16.5% had PI 1-2 and only 1% had PI > 2.

The prevalence of gingivitis was highest among current cigarette smokers (84.2%), whereas the prevalence of periodontitis was highest (17.6%) among past smokers, less among current smokers (8.4%) and least among non-smokers (7.4%). There was a significant difference in the prevalence of periodontitis with regard to cigarette smoking between subjects in the periodontitis and healthy groups (*p = 0.027*).

### Extent and Severity of Periodontal Diseases

The PI, GI, PD and CAL means are shown in Table 3. This Table shows that the healthy group demonstrates the lowest values of all examined parameters, whereas the AgP group shows the highest values. In addition, this Table shows that although the PI means of AgP and CP are comparable, the PD and CAL means for the AgP group are higher.

**Table 3:** The PI, GI, PD and CAL Means of the Study Population

VARIABLE	PI	GI	PD	CAL
	**MEAN ± SD**	**MEAN ± SD**	**MEAN ± SD**	**MEAN ± SD**

Healthy	0.37 ± 0.45	0.097 ± 0.34	1.86 ± 0.33	0.00 ± 0.00
Aggressive periodontitis	1.67 ± 0.52	1.96 ± 0.38	3.5 ± 0.37	1.7 ± 1.2
Chronic periodontitis	1.5 ± 0.35	1.6 ± 0.44	2.86 ± 0.38	1.16 ± 1.1
Gingivitis	1.34 ± 0.54	1.35 ± 0.45	2.26 ± 0.4	0.012 ± 0.14

Table 4 demonstrates that almost 30% of sites in periodontitis subjects aged ≥ 50 years exhibited CAL ≥ 3 mm and this extent dropped to almost 7% in younger individuals (20-29 years). CAL ≥ 6 mm was present in 11% of sites among subjects aged 40-49 years and dropped to almost 8% of sites among subjects ≥ 50 years old. The percentage of sites with PD ≥ 3 mm was highest among subjects aged 40-49 years and the lowest percentage was observed among subjects < 20 years old. As for disease severity, the highest mean GI, CAL and PD values were observed in the oldest age group (≥ 50 years).

**Table 4:** Extent and Severity of Periodontitis and GI according to Age

Variables		Age group (years)					
		**< 20**	**20-29**	**30-39**	**40-49**	**≥ 50**	

		**Mean (SD)**	**Mean (SD)**	**Mean (SD)**	**Mean (SD)**	**Mean (SD)**	**p-value**

**% of Sites with CAL (mm)**	≥ 3	0.0 (0.0)	0.8 (6.6)	3.3 (13.7)	9.7 (22.7)	26.7 (29.7)	< 0.0005
	≥ 4	0.0 (0.0)	0.8 (6.6)	3.3 (13.8)	9.3 (22.6)	24.0 (28.0)	< 0.0005
	≥ 5	0.0 (0.0)	0.4 (4.0)	2.0 (8.9)	6.7 (13.4)	13.2 (18.2)	< 0.0005
	≥ 6	0.0 (0.0)	0.1 (2.3)	0.5 (3.6)	3.1 (11.0)	2.9 (8.2)	< 0.0005
							
**% of sites with PD (mm)**	≥ 3	20.9(26.6)	28.3 (31.0)	41.7(37.0)	44.9(36.5)	55.1 (33.9)	< 0.0005
	≥ 4	0.0 (0.0)	0.9 (6.0)	2.3 (11.3)	5.6 (19.2)	9.3 (18.6)	< 0.0005
	≥ 5	0.0 (0.0)	0.3 (2.9)	0.5 (3.3)	3.4 (13.2)	4.4 (10.3)	< 0.0005
	≥ 6	0.0 (0.0)	0.1 (0.4)	0.1 (0.7)	0.7 (3.1)	0.5 (2.2)	0.001
							
**Disease Severity (mean)**	GI	1.1 (0.78)	1.1 (0.7)	1.3 (0.6)	1.2 (0.6)	1.5 (0.5)	0.001
	CAL	0.0	0.0 (0.3)	0.2 (0.7)	0.5 (1.3)	1.3 (1.5)	< 0.0005
	PD	2.1 (0.4)	2.2 (0.5)	2.4 (0.5)	2.5 (0.7)	2.7 (0.6)	< 0.0005

### Multivariate Analysis

In the multivariate analysis (Table 5), the only factors significantly associated with periodontitis were age, years of education, family history of periodontal diseases and frequency of tooth brushing. For each 1 year increase in age, the odds of having periodontitis increased by 20%. Years of education ≤ 12 years was associated with increased odds of having periodontitis by 5.5 times, compared to years of education > 12. The odds of having periodontitis increased almost 25 times among subjects who reported not brushing their teeth compared to subjects who reported regular tooth brushing. Subjects who reported the presence of periodontal disease among other members of their families had almost 5 times higher odds of having periodontitis than subjects who reported negative family history.

**Table 5:** Multivariate Analysis of Factors Associated with Periodontitis

Variables	OR	P-value
**Age (years)**	1.2	0.000
		
**Education (years)**		0.020
*≤ 12*	5.5	
*> 12*	1	
		
**Frequency of Tooth brushing**		0.024
*No*	24.9	
*Irregular*	2.6	
*Regular*	1	
		
**Family History**		0.033
*Yes*	4.8	
*No*	1	

## Discussion

This is the first study reporting the prevalence and risk indicators of three periodontal diseases among adults in the northern part of Jordan. The prevalence of AgP was 2.2%, of CP was 5.5% and of gingivitis was 75.8%. The comparison of the current results with previous studies is hindered by the use of different nomenclature and diagnostic criteria across studies. The prevalence of CP was lower than expected, possibly because most participants were young and educated, or because of the strict exclusion criteria. The prevalence of AgP was within the range reported in the US for whites (0.6%) and African Americans (2.8%) [6]. A study on Jordanian adolescents reported that deep pockets were found in only 0.29% of the study sample [13]. A higher prevalence of AgP (6.0%) has been recorded in Iraq [14] and in Brazil (5.5%),[15] but a lower prevalence (1.7%) was reported among Norwegian schoolchildren [14] than that obtained in the present study.

Most participants were 20-29 years old, probably reflecting the large proportion of young individuals in the Jordanian population or the possibility that most escorts were young. The lowest proportion of participants was ≥ 50 years of age, probably because many older individuals were patients (not escorts) and were not recruited. All AgP subjects were under 30 years, which is not surprising as it is universally accepted that AgP starts early in life in susceptible individuals. Many authors believe that age is not a risk determinant, but the life time disease accumulation [16]. In the multivariate analysis, for each one year increase in age the odds of having periodontitis increased by 20% and most subjects with CP were above 40 years of age, in agreement with other studies [15,17].

Our results demonstrated that most gingivitis patients were males, which may be attributed to poor attitude towards health and smoking. As mentioned above, AgP and CP were summed together into one "periodontitis group" when studying risk indicators (except age), because the number of AgP patients was low. The male to female ratio of periodontitis (1.6:1) is in agreement with other studies [18,19].

With regard to AgP, studies on Caucasians and Hispanics have demonstrated a greater female preponderance,[20] whereas studies involving Blacks demonstrated a greater prevalence in males. Albandar et al (2000) [19] reported a higher prevalence of AgP among females than males in Saudi Arabia with a ratio of 1.9:1. In a study in southern Brazil, AgP was distributed equally between males and females [21]. The reasons for these gender differences may be genetic factors, attitude toward oral health and dental-visit behavior [22].

The prevalence of gingivitis and periodontitis was higher in the low income than in the high income category, probably due to difficulty in affording dental treatment and oral hygiene aids. Difference in the prevalence of periodontitis according to SES was observed in other studies [23]. However, some studies have shown a weak association between SES and periodontitis after adjustment for oral hygiene and smoking [16,24].

In the present study, a low level of education was significantly associated with increased prevalence of periodontitis and the odds of having periodontitis increased by 5.5 times in subjects with ≤ 12 years of education. A higher prevalence of periodontitis among subjects with low education has been reported in Thailand [23]. In the USA, Borrell et al (2006) [25] reported that subjects with < high school education were 3 times more likely to have periodontitis than subjects with a higher level of education.

In the present study, subjects living in rural areas had a higher prevalence of gingivitis and periodontitis than subjects living in urban areas. Rural areas often have lower socioeconomic conditions and medical facilities than urban areas. Several studies have documented an association between area-based socioeconomic indicators and health outcomes [26]. Borrell et al (2006),[25] using data of the NHANES III, reported an association between periodontitis and neighborhood socioeconomic conditions.

A significantly higher prevalence of periodontitis was recorded among obese subjects than among normal weight subjects in the present study, which coincides with the results of the NHANES III in the USA [27]. Gingivitis was most prevalent among overweight subjects, although it was high in all BMI categories, with no clear pattern of association. A dose-dependent association between BMI and periodontitis was reported in Japan [28] after adjusting for age, sex, smoking and frequency of toothbrushing. Other studies [29] reported no significant difference in the prevalence of periodontitis between obese, overweight and normal subjects in males. Imbalance in the host immunity due to increased blood lipid and glucose levels in obese subjects may play a role in this association [30].

A higher prevalence of gingivitis and periodontitis was noticed among subjects who reported emergency dental visits, compared to subjects reporting regular dental visits, which is in agreement with reports in Brazil [21]. The lowest prevalence of gingivitis and periodontitis were observed among subjects reporting regular tooth brushing, whereas subjects reporting no tooth brushing had 25 times higher odds of having periodontitis. The effect of maintaining good oral hygiene on the periodontium is well documented [17,31]. In Accordance with this, our study demonstrated that most individuals with low PI (< 1) were periodontally healthy, whereas most people with gingivitis, CP and AgP had a high PI.

In this study, gingivitis was observed mostly in current smokers, probably indicating the strong and rapid effect of smoking on gingival health. The prevalence of periodontitis was also higher among smokers, in agreement with other studies [32]. Past smokers had a higher prevalence of periodontitis than current smokers; probably because the majority of past smokers were older in age and usually reported a long duration of smoking. Subjects who reported family history of periodontal disease had 5 times higher odds of having periodontitis than subjects with no family history. Positive family history of periodontal diseases has been reported previously [33,34].

This study has demonstrated that when challenged with similar amounts of plaque, people with AgP suffer a greater amount of attachment loss, compared to subjects with CP, indicating a higher susceptibility of the AgP group to periodontal destruction.

The greatest attachment loss and probing depth were present among older subjects (≥ 50 years) and that the extent of periodontal destruction increased with increasing age, within all categories of disease severity (i.e. CAL categories), indicating cumulative periodontal destruction in susceptible individuals [16]. When addressing the different severities of periodontal destruction an interesting finding was observed; as from CAL ≥ 5 mm the extent of disease decreased, indicating that more sites were affected by moderate periodontitis than by severe periodontitis; teeth affected by severe periodontitis may have been lost.

### Limitations to the work

1. The main limitation of this study was unavailability of a portable x-ray unit to the researchers. Numerous commercially available units exist, but almost all of them suffer serious safety shortages such as back scatter of the x-rays. They were evaluated by a specialist radiologist and the only "safe" portable x-ray machine could not be obtained by the researchers, despite repetitive attempts. This dictated the choice of places where subjects could be recruited; i.e. clinics and centers where a dental x-ray unit was present. In future studies, a full portable examination unit should be obtained.

2. Many of the variables investigated depended on patients' reports such as oral hygiene habits, smoking and other. Subjects' reports may not be true or accurate sometimes.

### Strength of the Study

The strength of the present study may be summarized in several points

1. The study sample was randomly selected from four centers in North Jordan; a large population in the Northern part of Jordan attend these centers.

2. Full clinical periodontal examination was performed for each subject.

3. Diagnosis of CP and AgP was based on, and strictly in accordance with, the criteria set by the AAP in 1999.

4. Diagnosis of periodontitis was confirmed radiographically using bitewing/periapical radiographs.

## Conclusions

It is concluded that 75.8%.of north Jordanians in this study had chronic gingivitis, 5.5% had CP and 2.2% had AgP. Age, education, frequency of tooth brushing and family history were significantly associated with increased risk for periodontitis. We recommend the onset of larger scale cross-sectional studies involving the whole country to investigate the prevalence of gingivitis and periodontitis. The risk indicators should be investigated in longitudinal studies to elucidate whether they are true risk factors.

## Competing interests

The authors declare that they have no competing interests.

## Authors' contributions

KA participated in the research design, generally supervised the work and wrote the manuscript. ZA carried out participant interview and examination, participated in data analysis and in writing the manuscript. YK put the research design, generally supervised the work and carried out all statistical analysis. All authors have read and approved the final manuscript.

## Pre-publication history

The pre-publication history for this paper can be accessed here:

http://www.biomedcentral.com/1472-6831/12/1/prepub
